# Genetic Diversity and Maternal Phylogenetic Relationships among Populations and Strains of Arabian Show Horses

**DOI:** 10.3390/ani13122021

**Published:** 2023-06-17

**Authors:** Mohamed Machmoum, Bouabid Badaoui, Daniel Petit, Agnès Germot, Moulay Abdelaziz El Alaoui, Ismaïl Boujenane, Mohammed Piro

**Affiliations:** 1 Veterinary Genetic Laboratory, Department of Medicine, Surgery, and Reproduction, Institut Agronomique Vétérinaire Hassan II, Rabat 10101, Morocco; m.piro@iav.ac.ma; 2 Biodiversity, Ecology and Genome Laboratory, Department of Biology, Faculty of Science, Mohammed V University in Rabat, Rabat 15062, Morocco; bouabidbadaoui@gmail.com; 3 African Sustainable Agriculture Research Institute (ASARI), Mohammed VI Polytechnic University (UM6P), Laâyoune 70000, Morocco; 4 LABCiS, University of Limoges, UR 22722, F-87000 Limoges, France; daniel.petit@unilim.fr (D.P.); agnes.germot@unilim.fr (A.G.); 5 Superior School of Technology, EST-Ibn Tofail University, Kenitra 14000, Morocco; moulayabdelaziz.elalaoui@uit.ac.ma; 6 Department of Animal Production and Biotechnology, Institut Agronomique et Vétérinaire Hassan II, Rabat 10101, Morocco; ismail.boujenane@gmail.com

**Keywords:** genetic variability, whole D-loop mitochondrial DNA, desert-bred, straight Egyptian, Polish Arabian, traditional Arabian horse classification

## Abstract

**Simple Summary:**

The Arabian horse is probably one of the most historic horse breeds worldwide. It has been selectively bred by the Bedouin tribes in the steppes of Arabia for over 2000 years. Bedouin tribes have created their own classification of strains based on maternal lines. The aim of our study was to investigate genetic differentiation at the level of (i) the current populations (Desert-Bred, Straight Egyptian and Polish Arabians) and (ii) the traditional Bedouin strains. Two hundred and eleven Arabian individuals, representing 12 strains, were sampled. Mitochondrial DNA D-loop analysis showed higher genetic diversity in the Egyptian and Polish Arabian populations than in the Desert-Bred. No genetic markers distinguishing the traditional strains were highlighted in this study, a fact probably due to the lack of interest in this component in modern breeding programs. Some Polish Arabian individuals, who could not be traced back to the Bedouin tribes, appeared to be genetically distinct from the other studied horses, requiring more in-depth study. This research, complemented by future SNP and Y-chromosome analyses, may provide a more accurate evaluation of the relationships between Arabian horse populations′ genetic and traditional classification.

**Abstract:**

Genetic diversity and phylogenetic relationships within the Arabian show horse populations are of particular interest to breeders worldwide. Using the complete mitochondrial DNA D-loop sequence (916 pb), this study aimed (i) to understand the genetic relationship between three populations, the Desert-Bred (DB), a subset of the Kingdom of Saudi Arabia (KSA), United Arab Emirates (UAE) and Bahrain (BAH), the Straight Egyptian (EG) and the Polish bloodline (PL), and (ii) to assess the accuracy of the traditional strain classification system based on maternal lines, as stated by the Bedouin culture. To that end, we collected 211 hair samples from stud farms renowned for breeding Arabian show horses from Nejd KSA, Bahrain, Egypt, Qatar, Morocco, UAE, and Poland. The phylogenetic and network analyses of the whole mitochondrial DNA D-loop sequence highlighted a great genetic diversity among the Arabian horse populations, in which about 75% of variance was assigned to populations and 25% to strains. The discriminant analysis of principal components illustrated a relative distinction between those populations. A clear subdivision between traditional strains was found in PL, in contrast to the situation of DB and EG populations. However, several Polish horse individuals could not be traced back to the Bedouin tribes by historical documentation and were shown to differ genetically from other studied Bedouin strains, hence motivating extended investigations.

## 1. Introduction

Arabian horses are a worldwide breed appreciated for their unparalleled beauty, human character, and versatility under saddle. All of these attributes are legacies of the Arab Bedouin tribes who lived across the steppes of Arabia for several centuries. The Bedouin tribes, who developed the breed, established specific requirements before the horse could be considered purebred (*Assil/Atiq*). Firstly, its pedigree (*Al-Nasab*) must be solely based on breeding from Arabia and free of any evidence of morphological or genealogical sign of impurity (*Hujna*, non-Arabian blood crossbreeding); therefore, it must be related to a recognized strain name (*Rasan*) and a sub-strain (*Marbat*). Secondly, its morphological features must match those of the breed (*Al-Muwasafat*). The strain names came from the original mares (or group of mares) that founded different families of *Asil/Atiq* horses centuries ago within foundational *Marbat* [1,2]. The transmission of these names was strictly maternal, justifying our analyses of mitochondrial sequences whose inheritances are also maternal.

However, it is historically established that the Arab Bedouins horses (Desert-Bred: DB), including Syrian, Bahraini and Saudi populations, were the sole ancestors of the Straight Egyptian (EG) Arabian and that they contributed largely to the creation of the Polish Arabian (PL) [3,4]. Moreover, EG stallions have impacted the PL population since the end of the 20th century. The transformation of the social model in the cradle regions largely impacted the DB population as well as the central role it had during the period of Bedouin nomadism and before mechanization and urbanization. However, the small size of the DB populations, the lack of extensive breeding programs and the limited uses reserved for them would argue in support of the conservation of an ancient phenotypic profile, which would logically be associated with the maintenance of a differentiated gene pool. The current DB stud farms are essentially devoted to the preservation of this great genetic legacy [1].

As for the EG horses, the background sources confirm that they were based on premium Bedouin horses from Arabia [1,3]. The manuscript of Abbas Pasha is widely considered as a reference of the main founders’ horses for the EG population. Indeed, it provides details on the origin and history of the founding horses concerning the Bedouin tribes [3,5]. The selection programs, which have been instituted mainly since the beginning of the 20th century in Egypt, USA and Europe, contributed to the development of this Arabian horse population. Its current morphological profile would be the result of environmental and selection criteria related to the undertaken breeding programs. Indeed, the increasing prosperity of the show competitions has oriented this selection in favor of the search for modern Arabian horses distinguished by their classical type, notably an exotic head with a dish-shaped profile, wide-set eyes, a high tail carriage and a marked charisma [6,7]. The modern PL population was likely produced, following the genealogical tables, by about 30 female and male founder lines originally from the steppes of Arabia in their majority [8,9]. Since the 19th century, Polish state stud farms have been conducting breeding programs that led to the production of a specific profile, the so-called “Polish type”, good, solidly structured and sound horses.

Microsatellite [10,11] and mtDNA D-loop [12] analyses indicated that the three Arabian populations (DB, EG and PL) showed high genetic diversity, reaching the levels recorded in Iranian and Syrian Arabian populations. The microsatellite approach also revealed that the genetic differentiation between the three populations was low to moderate, but significant and that the PL was closer to DB than to EG, confirming the historical documentation about the origin of the PL population cited above. As the DB population was an ancestor to both EG and PL, it was expected that the DB should have a higher genetic diversity than the other two, but microsatellite data revealed that this was not the case: PL harbored the highest value of heterozygosity [10]. As for maternal strains, the studies using SNP BeadChip [13,14] and mtDNA D-loop [14] did not find clear subdivisions based on the classification from maternal lineages.

Through the sequence analysis of DB, EG and PL Arabian populations, we aimed to first challenge the level of maternal genetic diversity in the Arabian horse populations as highlighted by previous studies and the significant differentiation between them [12,13]. It was also necessary to investigate the discrepancy between the predicted higher level of genetic diversity in DB and the low value revealed by the microsatellite approach. To go further, we should find common markers in terms of haplotypes and haplogroups between DB and EG from one side and DB and PL from the other side. Concerning the traditional strain based on maternal lines, we also aimed to verify whether this traditional system is supported by mitochondrial genetic data.

## 2. Materials and Methods

### 2.1. Population Samples

Hair samples were collected from 211 Arabian horses composed of three populations DB (n = 54), PL (n = 51) and EG (n = 106), all coming from stud farms localized in KSA, UAE, Qatar, BHR, Poland, Egypt and Morocco. All samples were unrelated from the mother’s side at least for three generations and had the phenotype of an Arabian horse breed, and were registered in a WAHO-recognized studbook. The geographical location of these stud farms is described in Supplementary Figure S1.

The studied horses were then regrouped into classical Arabian strains (*Rasan*) using their pedigrees and origins. Thus, 193 horses out of 211 were assigned to 12 strains: *Dahman*, *Hadban*, *Hamdani*, *Kuhailan*, *Obeyan*, *Saqlawi*, *Suwaiti*, *Subeyli*, *Al-Maanaqy*, *Al-Musannah*, *Al-Radba* and *Al-Tuwaisah*. The individual Polish horses derived from Szamrajówka, Ukrainka and Wołoszka dam lines, whose founders’ mothers could not be traced to the Bedouin tribes’ horses, were included as an unknown origin (PLS) (Supplementary Materials Table S1), and they have been described as “Saqlawi Polish Arabian” [8]. Otherwise, only strains with more than five individuals were included in the analysis.

### 2.2. DNA Extraction and Whole D-Loop Sequencing

Total genomic DNA was extracted from hair follicles using the PUREGENE^®^ DNA purification kit (QIAGEN, cat. no 158622, Hilden, Germany) following the manufacturer’s instructions. DNA quality was assessed with the NanoDrop 8000 spectrophotometer (Thermo Fisher Scientific, Waltham, MA, USA). The whole mitochondrial D-loop region was amplified using the pairs of specific primers tested previously by Khanshour and Cothran [14] in the upstream part (Hyper Variable Region 1) between sites 15440 and 16108 (Forward: 5′-AGCTCCACCATCAACACCCAAA-3′. Reverse 5′-CCATGGACTGAATAACACCTTATGGTTG-3′) and in the downstream part (HVR 2) between sites 16377 and 16642 (Forward: 5′-ACCTACCCGCGCAGTAAGCAA-3′. Reverse: 5′-ACGGGGGAAGAAGGGTTGACA-3′). The PCR reactions were performed for each part separately in a final volume of 25 μL using 100 ng genomic DNA, 20 μM of each primer, 5 μL 5× MyTaq Reaction Buffer and 0.2 μL MyTaq DNA polymerase (Bioline, London, UK, P/N: BIO-21105). The PCR conditions comprised initial denaturation at 95 °C for 3 min, 35 cycles at 95 °C for 15 s, annealing at 55 °C for 15 s and extension at 72 °C for 20 s, followed by a final extension at 72 °C for 1 min. The reactions were carried out in a Veriti Thermal Cycler (Applied Biosystems, Foster City, CA, USA). Before sequencing PCR, the PCR products were enzymatically cleaned up using the ExoSAP-IT reagent (Thermo Fisher Scientific). Sequencing reactions were performed using BigDye Terminator v3.1 Ready Reaction Cycle Sequencing Kit (P/N: 4337455) with an ABI PRISM 3130XL Genetic Analyzer (Applied Biosystems) using the POP-7 polymer (P/N: 4393708). mtDNA sequences were analyzed using Sequencing Analysis Software version 5.3.1 (Applied Biosystems, P/N: 4360967) and assembled using DNA Dragon Sequence Assembler v.1.6.0 (Sequentix-Digital DNA Processing, Warnow, Germany). Alignment and editing of all sequences were carried out by MEGA software [15] using the horse mtDNA GenBank sequence X79547 as a reference [16].

### 2.3. Data Analyses

Haplotype sequences included in this study were registered into the National Center for Biotechnology Information (NCBI) GenBank database available at http://www.ncbi.nlm.nih.gov/the accession numbers NCBI: MZ735748–MZ735958 (accessed on 6 August 2021). The genetic variations within populations were evaluated by using the DNAsp 5.10.1 software [17]. The following diversity parameters were calculated: the number of haplotypes (NHap), the haplotype diversity (HD), the nucleotide diversity (Pi), the number of polymorphic sites (NPs) and the average nucleotide differences (K). Additionally, four neutrality tests were performed: Fu and Li F*, Fu and Li D* [18], Fu Fs [19] and D of Tajima [19] using DNAsp for each population.

To estimate the genetic differentiation between studied populations, the pairwise FST was calculated using the Kimura 2-parameter model with 1000 permutations and was carried out with Arlequin 3 [20]. For the interpretation of pairwise FST results, we followed the suggestion indicating that a value between 0–0.05 shows little genetic differentiation; a value between 0.05 and 0.15 shows moderate differentiation; a value between 0.15 and 0.25, indicates great differentiation; while a value above 0.25 indicates high genetic differentiation [21,22,23].

The phylogenetic analysis of the haplotype using the whole D-loop sequence was conducted with MEGA software version 10.0 [15]. The phylogenetic tree was constructed using the Maximum Likelihood (ML). The best model according to the lowest Bayesian Information Criterion corresponded to the Tamura 3-parameter model, including Gamma distribution and Invariant sites (G + I). The individuals corresponding to each haplotype were represented by colored circles depending on the tested populations and strains, and the haplogroups were assigned as defined by Achilli et al. [24]. The donkey (Equus asinus) mtDNA sequence (GenBank: NC_001788) was used as an out-group [25]. Using Network software 10.1.0, the median-joining network (MJ network) of the whole D-loop sequence haplotypes was constructed [26]. To avoid reticulations, the Star Contraction option was used to reduce the large data set, and the maximum parsimony (MP) calculation procedure based on the Neighbor-Joining method was used to remove unnecessary vectors and median links. Haplotypes in the MJ network were shown by a color code linked to tested populations or strains, allowing visualization of their proportions depending on the individual frequencies in each haplotype.

To verify if there was a significant variation between haplogroup compositions in maternal lines, two separate approaches were applied using PAST 2.17c software [26]. At the level of individuals, the analysis of similarities (ANOSIM) test was used by adopting the Chord distance index, and the related *p*-value was deduced from a set of 9999 permutations. At the level of strains, the chi-square (χ2) test was applied for each combination of studied lines, and the *p*-value was calculated by the Monte Carlo test.

To visualize the proximities between the studied strains, a Cluster analysis was conducted with the Chord distance index implemented in PAST 2.17c, and the significance of the branches was assessed by 1000 bootstrap iterations.

Discriminant analysis of principal components (DAPCs), implemented in the adegenet package for R [27], was applied to the mtDNA data set to examine the population genetic structure and to assess the degree to which tested populations differed from each other using supervised clustering. The DAPC approach is proposed to optimize the separation of individuals into predefined groups based on a discriminant function of principal components, to assign individuals and to obtain the membership probability, which presents the overall genetic background of an individual. This method was also applied to figure out the overlap between the traditional and genetic classifications, considering both the population and strain of animals for which this information was available.

Using the gl.manova function from the DartR package, a multivariate analysis of variance (MANOVA) was conducted on genetic distance data, with a focus on horse populations and horse strains. The Phi-statistic was determined to estimate the proportion of genetic differentiation among populations relative to overall genetic differentiation.

## 3. Results

### 3.1. Haplotypes and Haplogroups

**Figure 1:**
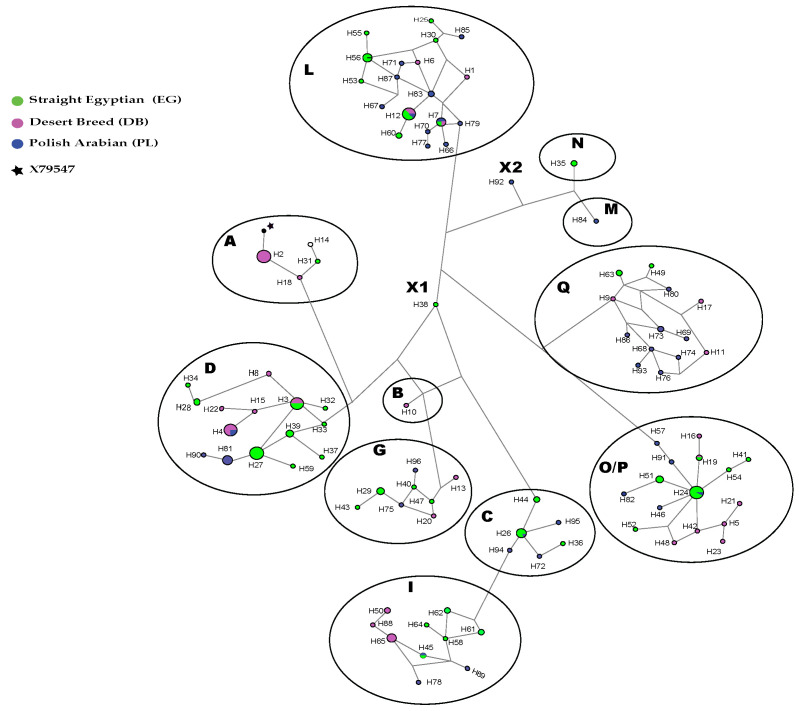
Median-joining network based on 916 bp of the mitochondrial D-Loop representing 211 horses within 96 haplotypes. The haplogroups (A, B, C, D, G, I, L, M, N, O/P, Q) were named as defined by Achilli et al. [23]. X1 and X2 correspond to unclassified haplogroups. Each population is shown by color, and the size of nodes is proportional to the haplotype of different populations. The reference horse sample X79547 is labelled with a star in haplogroup A.

Among the 211 individuals, 96 haplotypes were identified from the whole mtDNA D-loop (916 bp) using DNAsp [17]. Sixty-nine polymorphic sites were detected, and the haplotypes differed from each other by one to 14 variations (Supplementary Table S2). Supplementary Figure S2 shows the consensus Neighbor-joining tree of the 96 haplotypes found in our tested populations. It appeared that they were not clearly differentiated in separated clusters and that the majority of haplogroups were shared between the three populations. Eighteen haplotypes were only found in DB, thirty-eight in EG and thirty-one in PL. The DB shared two haplotypes with EG and one with PL. Five haplotypes were shared by the EG and PL. Two haplotypes (H7 and H12) were in common among the three studied populations. The median-joining network (MJ network) based on 916 bp of the D-loop is shown in Figure 1. Each haplotype is shown by the proportion of different populations included in it. The haplogroups among populations were differentially distributed (*p* < 0.05) according to the Chi-2 test (Supplementary Materials Figure S3a).

Twelve haplogroups: A, B, C, D, G, I, L, M, N, O/P and Q according to Achilli’s classification [24], were identified. In DB, haplogroup A showed the highest frequency, while haplogroups C, I, M and N were absent. In EG, the haplogroups D and O/P were largely present, while B and M haplogroups were absent. In PL, the A, B and N haplogroups were absent. However, two haplotypes (H38 for EG and H92 for PL) were not assigned to any of Achilli’s haplogroups and were identified as haplogroups X1 and X2, respectively. However, as shown in Supplementary Figure S2, the haplogroup X1 was close to the B and G haplogroups, while X1 was close to M and N. As also shown in Supplementary Figure S2, the three tested populations were represented together in six haplogroups (D, G, L, O/P and Q). The EG and PL populations shared the haplogroups C and I. The haplogroups B, M and N were represented in DB, PL and EG individuals, respectively. The haplotypes O/P, D and L were the most shared between the three populations and represented 22%, 17% and 16% of the total samples, respectively. The DB was the least variable among tested populations, with eight haplogroups (A, B, D, G, L, O/P, Q).

**Figure 2:**
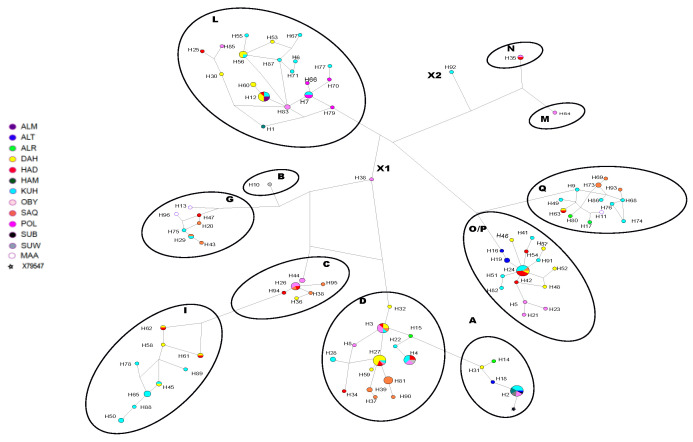
Median-joining network representing 193 horses within 93 haplotypes. The haplogroups (A, B, C, D, G, I, L, M, N, O/P, Q) were named as defined by Achilli et al. [24]. X1 and X2 correspond to unclassified haplogroups. Each strain is shown by color, and the size of nodes is proportional to the haplotype of different strains. The horse reference sample X79547 is labelled with a star in haplogroup A.

Supplementary Figure S4 shows the consensus Neighbor-joining tree of the 93 haplotypes found in the 193 individuals that were assigned to strains. Eighteen haplotypes were identified in at least in two strains (haplotypes 1, 6, 7, 8, 11, 12, 14, 15, 22, 23, 24, 26, 31, 40, 43, 46, 65, 78), and then all the rest were only found in one of the tested strains. The most variable strains that had their individuals present in the majority of the clades were *Kuhailan*, *Dahman*, *Saqlawi*, *Hamdani*, and *Obeyan* with 36, 27, 19, 12 and 10 haplotypes, respectively. The *Al-Musannah* and *Subeyli* strains were represented uniquely by one haplotype each, H1 and H6, respectively. The MJ network based on 916 bp of the D-loop for strains is shown in Figure 2. The haplogroups among strains were differentially distributed (*p* < 0.05) according to the Chi-2 test (Supplementary Materials Figure S3b).

Eight strains shared two or more haplogroups, and individuals from the same strain were found in separate haplogroups. The *Saqlawi* and *Kuhailan* were the most variable strains, with individuals distributed across nine and eight haplogroups, respectively. The unknown group (PLS) and *Al-Musannah* strain were only present in haplogroup L, and they were close to the *Dahman*, *Hadban*, *Hamdani*, *Kuhailan*, *Saqlawi* and *Suwaiti*.

### 3.2. DNA Sequence Polymorphism and Tests of Neutrality

The polymorphism analysis using the whole D-loop sequence (Table 1) showed the presence of 96 haplotypes from the 69 polymorphic sites in 211 horses from all tested populations. The number of haplotypes (NHap) per population ranged from 23 in DB to 45 in EG. The number of polymorphic sites (NPs) ranged from 44 in DB to 55 in EG. For all individuals together, the value of the haplotype diversity (HD) was about 0.964. It varied among populations from 0.822 in DB to 0.985 in PL. The lowest value of nucleotide diversity (Pi) was found in DB and EG (0.009). The calculated neutrality tests Fu and Li F* and D* among the three populations indicated no significant values. On the contrary, Fu’s Fs revealed that the three populations were significantly out of neutrality.

The mtDNA polymorphism analysis results concerning the strains are shown in Table 2. Eighty-six haplotypes (NHap) from 67 polymorphic sites (NPs) were observed for the 183 horses assigned to the seven retained *Rasans* (with at least five individuals). The *Kuhailan* was the most variable strain with NHap:36, followed by *Dahman* (NHap:23) and *Saqlawi* (NHap:21). *Suwaity* and *Hamdani* showed the lowest number of haplotypes (three and five, respectively). As the number of haplotypes by strain is strictly proportional to the size of the strains (*p*-value of Pearson < 10^−6^), the use of this data is limited and will not be considered in the following.

### 3.3. Genetic Differentiation between Populations and between Strains

#### 3.3.1. Population-Level

In the first step, discriminant analysis of principal components was undertaken to investigate the relationships between the populations. The clusters in DAPC were defined by *a priori* assumptions of population membership (K = 3) and strain membership (K = 8). The number of retained principal components was defined using the α-score optimization proposed by Jombart et al. (2010) [27], resulting in 30 PCs retained as input to DA, and cumulatively explaining about 99% and 98% of the total genetic variability for horse populations and strains, respectively.

**Figure 3:**
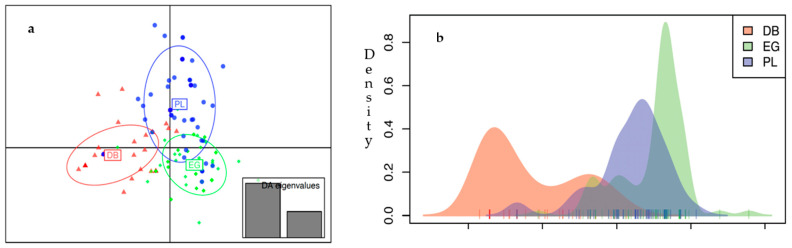
Distribution of individuals based on discriminant functions at the population level. (**a**) Individuals are represented by dots, and Arabian populations are shown using 95% inertia ellipses depicted around individuals based on the first two discriminant functions. (**b**) Density plot of populations of individuals based on first discriminant function.

The population structure deduced from DAPC revealed a clear genetic differentiation between the three tested populations with a narrow overlap among them (Figure 3a). The overlap zone between PL and EG was relatively wider than between PL and DB, while it was negligible between DB and EG. In addition, the first discriminant function scatter plot showed density variations along this axis indicating the level of internal population density (Figure 3b). All genotyped individuals are presented in Supplementary Figure S5. Each line represents one individual, and the heat color represents their membership probability, provided by DAPC, of being assigned to the predefined populations. The average assignment probability was 81.10%. The highest population assignment was the EG population (92.53%), while the population assignment percentage for DB and PL was 75.26% and 74.51%, respectively.

Pairwise FST used to explore the genetic relationships among the populations confirmed that the Polish bloodline was closer to the Straight Egyptian than to the Desert-Bred (Table 3).

#### 3.3.2. Strain Level

**Figure 4:**
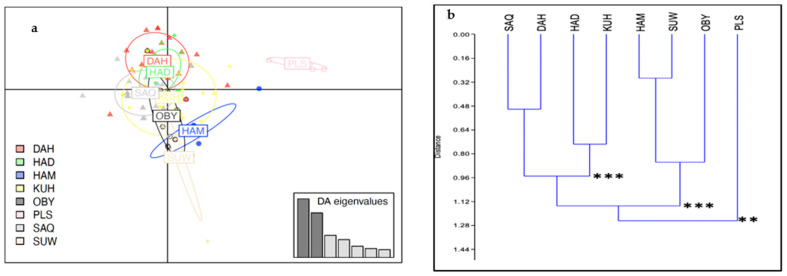
Distribution of individuals based on discriminant functions at the strain level. (**a**) Individuals are represented by dots, and Arabian horse strains (*Rasans*) are shown using 95% inertia ellipses depicted around individuals based on the first two discriminant functions. (**b**) Cluster analysis of strains with the Chord index as a measure of distance using PAST 2.17c [26]. The significances were deduced from bootstrap iterations, with *p* ** < 0.01, *p* *** < 0.001.

A DACP analysis was conducted on the strains in parallel with the population approach. Figure 4a presents the projection on the two discriminating factors and indicates some overlapping between these strains, except for the PLS group, which is separated from the rest. This is also illustrated by the first discriminant function scatter plot, which showed density variations of internal strain density (Figure S6).

To complete this analysis, the matrix with the number of individuals of each haplogroup per strain was submitted to a Cluster analysis (Figure 4b). The statistical significance of the clustering deduced from the Chi-2 test revealed four groups (SAQ-DAH, HAD-KUH, HAM-SUW-OBY, and PLS).

#### 3.3.3. Population and Strain Level Combination

**Figure 5:**
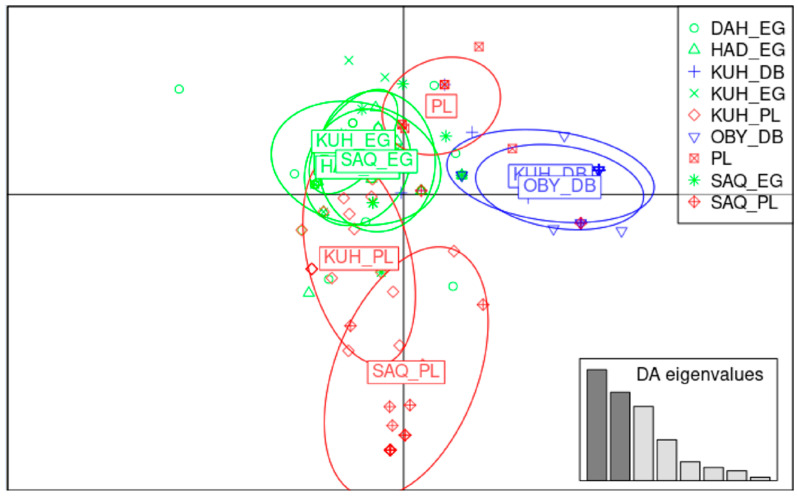
Distribution of individuals based on the first two discriminant functions performed on both Arabian horse populations and strains. Individuals are represented by dots and shown using 95% inertia ellipses depicted around individuals. DB in red, EG in green, and PL in blue.

To investigate how the strains are distributed within the populations, a new DAPC was conducted. Given the strain sizes within each population, only two strains were retained in the DB, four in the EG, and three in the PL. It showed that the strain envelopes in DB and EG overlapped or merged, while the PL population strains were separated. In other words, it seems not possible to distinguish the strains within the EG and DB populations, while certain strains in the PL population are clearly distinct (see Figure 5 below). 

Following the MANOVA, the Phi-statistics associated with the proportion of genetic differentiation among populations relative to the total genetic differentiation was found to be 0.07 for horse populations and 0.17 for horse strains. The corresponding *p*-values were less than 10^−4^, indicating a significant difference from what would be expected by chance. These Phi-statistic values suggest a moderate level of differentiation among the horse populations and strains under investigation. Moreover, the variance coefficients provided additional evidence of the impact of these factors on the overall variability in the data. The variance coefficient for horse populations was calculated as 65.8, while the coefficient for horse strains was 18.3. These relatively high coefficients indicated that both horse populations and strains contributed significantly to the observed variability in the genetic distance data. Overall, the Phi-statistics and variance coefficients indicated that there is moderate but significant differentiation among horse populations and strains, which is supported by the gl.manova analysis results. These results imply that both variables significantly contribute to the explanation of the genetic variation found in the data.

## 4. Discussion

This study used the mtDNA control region to highlight the genetic variability of DB, EG and PL Arabian horse populations, including the strain classification system. It has been proved that the whole mtDNA D-loop for genetic diversity analysis is more robust and powerful than using the hyper-variable region 1 (HVR1) alone for this purpose [12,28,29]. The maternal genetic diversity reported herein for the studied Arabian population is comparable to those reported by some previous studies [12,14,30,31,32]. The high number of haplotypes found in the three populations was comparable to that found in Arabian horses evaluated by Guastella et al. [31] and in Syrian Arabian horses by Khanshour and Cothran [12]. This large diversity in the Arabian horse population could be interpreted as a testimony of multiple origins in the maternal lineages of domestic horse breeds, as reported by several studies [14,33,34,35,36]. Additionally, several individuals in our samples belonged to haplogroup A, which, according to the Cieslakc classification, correlates with haplogroup D, which is one of the rare and ancient haplogroups that can be traced back to the Bronze Age [24,36]. The significant genetic diversity of the Arabian sequences confirms the heterogeneous origin of the breed. Regarding the undescribed haplogroups X1 and X2 found in two individuals, we interpret their presence as rare variants not retrieved in Achilli’s et al. work [24]. Given their position in the phylogeny, they could represent ancestral lines maintained in an Arabian breed.

As mentioned in the introduction, it has been established that the DB horse, developed by Bedouins under harsh climatic conditions in the steppes of Arabia, is the origin of all modern Arabian horse populations, including the EG and most PL lines [4,37,38].

Based on the pedigree analysis from the stud books data, several founding broodmares of the PL and EG populations were brought from the Arab region (Gazella db, Sahar db, Melcha db, Rodania db, Milordka db, Sherife db, Semrie db, Adjuze db, Ferida db) [30,36]. So, it was expected that DB had higher genetic diversity than both EG and PL. To test this hypothesis, three approaches were undertaken. They consisted in comparing the various indices of genetic diversity, the existing haplotypes, and the distribution of haplogroups among populations.

The study of haplotype distribution, based on the consensus NJ tree, showed that 18 haplotypes were only found in the DB individuals, while 38 and 31 haplotypes were found in the EG and PL individuals, respectively. The same trend was retrieved for the average number of nucleotide differences in DB, EG and PL populations. On the other hand, the number of haplotypes and haplotype diversity showed lower values in the DB population than in the EG and even more so in the PL. The number of polymorphic sites was intermediate in the PL population compared to the EG with the highest value and the DB with the lowest. The MJ network confirmed this result, where DB individuals were present in only eight haplogroups, while both PL and EG populations were represented in nine and 10 haplogroups. All of these data confirmed the expected high genetic diversity of the DB population, supporting the previous finding by microsatellite analyses [10].

The interpretation of these data is an open question. Despite the sampling within the DB population being performed based on the “*Rasan*”, there is no guarantee that all the diversity was captured, which may explain the relatively low genetic diversity of the DB. In addition, the current estimated size of the EG and DB populations between 3–10% of the global Arabian horse population would be far less than that of the PL population. Despite the exact size of the respective populations, this effect remains a possible cause of the low DB genetic diversity due to allele loss and genetic erosion. In addition, it is possible that the DB population has fallen below the critical minimum for survival, resulting in a bottleneck effect, explaining the low level of genetic diversity.

Regarding the origin of the PL population, our analysis showed that PL shared one and five exclusive haplotypes with DB and EG, respectively. We cannot exclude that some haplotypes have disappeared or were not sampled in our study, but the most likely hypothesis is that the majority of the maternal contribution for the PL population was Egyptian, although the possibility of the contribution of DB stallions is not excluded. However, the microsatellite data indicated a closer genetic relationship between PL and DB; this suggests that the DB stallions participating in the breeding program of the PL had a major impact on the genetic pool of the PL population [10]. An SNP analysis could give an important view on the global relationships between the three populations. The important variability observed in EG and PL populations could be due to our samples’ large variety of dam lines. In fact, 12 and 15 dam lines were represented in the EG and PL populations, respectively. Remer et al. [38] recently published a study in which the lineages of Arabian horses were tracked using the Y chromosome. It would be very beneficial to combine Y chromosome DNA with mtDNA to better understand the genetic connections between the various Arabian populations

According to history, the traditional Bedouins tribe’s Arabian classification was based on the notions of strain (*Rasan*), which are transmitted through the maternal lines, and sub-strain (*Marbat*), both constituting the identity of pure Arabian (*Assil/Atiq*) individual horses [4,39]. Mostly, losing strain information means a complete loss of identity. Because of the lack of detailed data concerning the notion of sub-strains (*Marbat*) of our samples, we limited our study to the strains.

To highlight the genetic structuring between strains, the low sample size of the strains *Maanaqy*, *Subeyli*, *Al-Musannah*, *Al-Radba* and *Al-Tuwaisah* made their characterization difficult. However, the control region mtDNA analysis findings showed that four groups of strains were identified (*Saqlawi-Dahman*, *Hadban-Kuhailan*, *Hamdani-Suwaiti*-*Obeyan* and PLS group) when the population level was not considered. It should be noted that only *Suwaiti* and *Saqlawi* strain individuals were found to belong to haplogroups B and M, respectively, which may support the genetic reality of these strains. In contrast, all seven strains were represented in haplogroup A. The possibility of some discrimination between strains in the Syrian Arabian horse population was previously mentioned [13]. However, our analysis showed that the influence of populations is stronger than that of strains in the case of DB and EG: we were not able to discriminate strain in these two populations. Regarding the PL population, it was unexpected that certain strains could be distinguished given that this population mainly derived from DB and EG populations. In the case of the unknown group (PLS), it was the only group represented in haplogroup L. Moreover, we noticed previously the impossibility of tracing the founders’ dams of this group back to Bedouin tribes’ horses, as was the case with the other founders’ Polish dam lines in our study. Indeed, the dam lines *Szamrajówka*, *Ukrainka*, and *Wołoszka* have had their origins traced back not far away from the stocks of Sławuta stud [8]. As for the distinction between *Saqlawi* and *Kuhailan* strains observed in the PL population, the explanation still needs to be determined. For a more detailed and objective interpretation of the origin of these Polish Arabian horses using Y sequences and SNP data, it is necessary to integrate the human population history’s results and the European/Asian migrations of peoples.

Hence, when we consider the reality of strains between populations, a low number of strain markers appears since the population effect dominates the strain effect. However, our phylogenetic results and DAPC did not reveal a clear distribution that fits strain subdivisions. This finding is consistent with some previous works [14,28,33]. Indeed, the strains are very ancestral, they go back to the seventh century at least, while the differentiation of the populations was made centuries later: from the nineteenth century for EG and almost after the World Wars for the PL. Since then, the selection performed in each population with specific programs, and the negligence of the strain system in this different selection, mainly in the PL population, have erased more or less their genetic characteristics. This system is more accurately tracked and documented in DB and EG than in the PL. Overall, according to the culture of Bedouin tribes, the strains and the sub-strains (*Rasan*-*Marbat*) both constitute the identity of pureblood Arabian horses (*Assil/Atiq*) [4,39]. Therefore, the lack of complete and reliable data on this traditional system questioned the possibility of any rational assessment of its usefulness.

## 5. Conclusions

Based on the whole mtDNA D-loop sequence, the present study confirmed the large genetic diversity of the Arabian horse populations and illustrated the genetic differences between DB, EG, and PL. Even though the DB is at the origin of EG and PL, it has been observed that it has the lowest genetic diversity, which may be due to genetic erosion related to the low population size. The maternal lineages of the PL population would be mostly of EG origin, and we hypothesize that a major paternal part could come from Desert-Bred ancestors, which would fit the results of microsatellite markers. Once again, this point would need to be investigated by Y chromosome analysis. A special mention must be stated about the maternal lineage of the PLS group, which needs to be investigated more deeply, as no connection was found with the Desert-Bred individuals of our sample. Concerning the traditional strain system of the Bedouins, there are only some elements in the mtDNA, but it is evident that the differentiation of the populations has erased more or less the genetic markers of the strains. Further, detailed studies including both strain and sub-strain (*Rasan-Marbat*) data as well as SNP data may shed additional light on the relationship between genetics and the traditional Bedouin classification system.

## Figures and Tables

**Table 1:** DNA sequence polymorphism and tests of neutrality in Desert-Bred, Straight Egyptian and Polish Arabian populations.

Pop.	N	NHap	NPs	HD (SD)	Pi (SD)	K	Fu Li’s D *	Fu Li’s F *	Fu’s Fs	T^h^
DB	54	23	44	0.822 (0.050)	0.009 (0.010)	8.372	0.167	−0.071	−3.011 **	−0.450
EG	106	45	55	0.925 (0.018)	0.009 (0.011)	11.553	0.758	0.688	−10.580 ***	0.309
PL	51	39	52	0.985 (0.009)	0.011 (0.013)	12.940	0.641	0.665	−16.969 ***	0.412
All	211	96 ^a^	69	0.964 (0.006)	0.013 (0.013)	11.780	0.150	0.118	−61.499 ***	−0.171

Pop.: population. N: number of individuals. NHap: number of haplotypes. NPs: number of polymorphic sites. HD: haplotypic diversity. Pi: nucleotide diversity. K: average number of nucleotide differences. SD: standard deviation. Fu, Fu Li’s, Fu’s Fs and T^h^: neutrality tests. ^a^ The NHap value for all strains (96) is lower than the sum of the NHap calculated for each one separately due to the share of common haplotypes between strains. *p* * < 0.05, *p* ** < 0.01, *p* *** < 0.001.

**Table 2:** Genetic diversity measures for Arabian horse strains (*Rasans*).

Strains	N	NHap	NP_S_	HD (SD)	Pi (SD)	K
*Dahman (DAH)*	38	23	38	0.936 (0.029)	0.012 (0.002)	11.030
*Hadban (HAD)*	24	14	44	0.833 (0.077)	0.011 (0.002)	10.395
*Hamdani (HAM)*	9	5	29	0.806 (0.120)	0.011 (0.018)	9.778
*Kuhailan (KUH)*	59	36	50	0.948 (0.018)	0.013 (0.002)	12.252
*Obeyan (OBY)*	19	11	22	0.924 (0.037)	0.007 (0.001)	7.053
*Saqlawi (SAQ)*	29	21	51	0.963 (0.023)	0.013 (0.013)	12.986
*Suwaity (SUW)*	5	3	14	0.700 (0.218)	0.007 (0.002)	6.200
All	183	86 *	67	0.961 (0.007)	0.013 (0.013)	11.820

N: number of individuals. NHap: number of haplotypes. NPs: number of polymorphic sites. HD: haplotypic diversity. Pi: nucleotide diversity. SD: standard deviation. K: average number of nucleotide differences. * The NHap value for all strains (86) is lower than the sum of the NHap calculated for each one separately due to the share of common haplotypes between strains. The strain names in italics correspond to their Arabic names.

**Table 3:** Pairwise FST statistics among Desert-Bred, Straight Egyptian and Polish Arabian horse populations.

	DB	EG	PL
DB	-	-	-
EG	0.013	-	-
PL	0.015	0.005	-

## Data Availability

The D-Loop sequences were deposited in the GenBank (NCBI) under the accession numbers MZ735748 to MZ735958.

## Supplementary Materials

The following supporting information can be downloaded at: https://www.mdpi.com/article/10.3390/ani13122021/s1, Figure S1: Geographic distribution of the studied Arabian horse sample; Figure S2: Consensus Neighbor-Joining tree of 96 mtDNA haplotypes found in Arabian horse populations; Figure S3: Chi-2 test results for Arabian populations and strains; Figure S4: Consensus Neighbor-Joining tree of 93 mtDNA haplotypes found in Arabian horse strains; Figure S5: Probability of membership of each individual in the studied populations; Figure S6: Density plot of Arabian horse strains, based on the first discriminant function. Table S1: Dam lines of Polish Arabian origin and their known strains; Table S2: Arabian horse haplotype sequences of mtDNA D-loop hypervariable region compared to the reference sequence GenBank X79547.
