# Supplementary material for: Genetic Diversity and Maternal Phylogenetic Relationships among Populations and Strains of Arabian Show Horses
Source: Animals (Basel). 2023 Jun 17;13(12):2021. doi: 10.3390/ani13122021 (PMC10295422; doi:10.3390/ani13122021)
Supplement: Supplementary file 1 [file animals-13-02021-s001.zip › animals-2373637-supplementary.pdf]

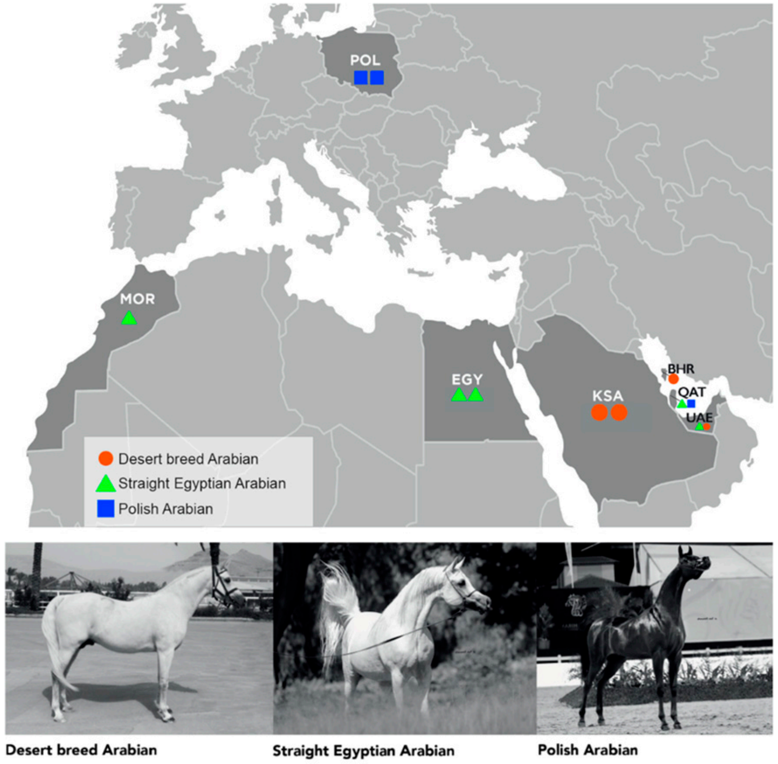

Figure S1 : Geographic distribution of the studied Arabian horse populations sampling

Supplementary Table S1. Dam lines of Polish Arabian origin and their known strains

**Dam line (year of birth)**	**Origin**	**Strain (substrain)[1]**
Adjuze db (1869)	Imp from Arabia in 1870 to Tiaret Stud (France)	Kuheilan
Bent-El-Arab db (1880)	Imp from Arabia in 1885 to Babolna Stud (Hungary)	Kuheilan
Ferida db	Imp from Arabia in 1845 to Jarczowce Stud (Poland)	Maanaqy (Sbaili)
Mlecha db	Imp from Arabia in 1845 to Jarczowce Stud (Poland	Kuheilan (Dajani)
Rodania db (~ 1869)	Imp from Arabia in 1881 to Crabet Park Stud (GB)	Kuheilan (Rodan)
Sahara db ~	Imp from Arabia in 1845 to Jarczowce Stud (Poland)	Kuheilan (Moradi)
Selma db (~1865)	Imp from Arabia in 1894 to Crabbet Park Stud (GB)	Hamdani (Simri)
Semrie db (1896)	Imp from Arabia in 1896 to Babolna Stud (Hungary)	Kuheilan
Scherife db (1896)	Imp from Arabia in 1902 to Babolna Stud (Hungary)	Kuheilan
Milordka (~1816)	Unknown Origin to Slawuta Stud (Poland)	Saqlawi[2]
Wołoszka (~1810)	Unknown Origin to Slawuta Stud (Poland)	Saqlawi[2]
Szamrajówka (~1816)	Unknown Origin to Slawuta Stud (Poland)	Saqlawi[2]
Ukrainka (~ 1815)	Unknown Origin to Slawuta Stud (Poland)	Saqlawi[2]

db : desert breed
[1] : Information from studbooks
[2] : Strains reported by Skorkowski (1972)

Supplementary Table S2. Arabian horse haplotype sequences of mtDNA D-loop hypervariable region compared to the reference sequence GenBank X79547. Haplotypes are listed according to an order suggested by a neighbor-joining algorithm using MEGA X. Black spot indicate concordance with the X79547 sequence.

Haplotypes	Haplogroups	Positions of nucleotide substitutions relative to Genbank X79547 as references sequences of horse																																																																		
		15461	15462	15463	15468	15502	15506	15510	15533	15552	15553	15564	15565	15569	15570	15571	15572	15603	15617	15618	15627	15634	15635	15671	15677	15688	15694	15708	15734	15738	15739	15745	15774	15775	15777	15778	15779	15794	15795	15806	15837	15838	15839	15847	15911	15924	15942	15953	15963	15975	15999	16005	16006	16008	16023	16339	16361	16400	16406	16475	16480	16497	16499	16510	16513	16523	16524	16615
	X79547	T	C	G	C	A	C	C	C	G	G	A	T	C	T	G	T	A	A	T	G	G	T	C	G	G	A	C	C	C	A	C	C	A	A	C	A	A	C	C	C	C	C	A	A	C	T	A	T	T	A	A	T	A	A	T	T	C	G	C	A	A	T	T	T	T	C	A
H1	L	.	T	.	.	.	.	.	.	.	.	.	.	.	C	.	.	.	.	.	.	.	.	.	.	G	.	.	.	.	.	.	.	.	.	.	.	.	.	.	.	.	.	.	.	.	.	.	.	.	.	.	.	.	.	.	.	.	.	.	.	.	.	.	.	.	.	.
H2	A	C	.	G	.	T	.	.	.	.	A	.	.	.	.	C	.	.	G	.	.	.	.	.	.	.	.	.	.	.	T	.	.	.	.	.	.	.	.	.	.	T	T	.	.	G	.	.	.	.	.	.	.	.	.	.	.	.	.	.	.	.	.	.	.	.	.	.
H3	D	.	.	.	.	.	.	.	.	.	.	.	.	.	C	.	.	.	.	G	.	A	.	.	.	.	.	.	.	.	.	.	.	.	.	.	.	G	.	.	.	.	.	.	.	.	.	.	.	.	.	.	.	.	.	.	.	.	.	.	.	.	.	.	.	.	.	.
H4	D	.	.	.	.	.	.	.	.	.	.	.	.	.	C	.	.	.	.	.	.	.	.	.	.	.	.	.	.	T	.	.	.	.	.	.	.	G	.	.	.	.	.	.	.	.	.	.	.	.	.	.	.	.	.	.	.	.	.	A	.	.	.	.	.	.	.	.
H5	O/P	.	.	.	.	.	.	.	.	.	.	.	G	.	.	.	.	.	.	.	.	.	G	C	.	.	.	.	T	.	T	G	.	.	G	.	.	.	.	.	.	.	.	.	.	G	.	.	.	.	.	.	G	.	.	.	.	.	.	A	.	.	.	.	.	.	.	G
H6	L	.	T	G	.	T	.	.	.	.	A	.	.	.	.	C	A	.	G	.	.	.	.	.	.	.	.	.	.	.	T	.	.	.	.	.	.	.	.	.	.	T	T	.	.	G	T	.	.	.	.	.	.	.	.	.	.	.	.	.	.	.	.	.	.	.	.	G
H7	L	C	.	G	.	T	.	.	.	.	.	.	.	.	.	C	.	.	G	.	.	.	.	.	.	.	.	.	.	.	T	.	.	.	.	.	.	.	.	.	.	T	T	.	.	G	T	.	.	.	.	.	.	.	.	.	.	.	.	.	.	.	.	.	.	.	.	G
H8	D	.	T	.	.	.	.	.	.	.	.	.	.	.	C	.	.	.	.	G	.	A	.	.	.	.	.	.	.	.	.	.	.	.	.	.	.	G	.	.	.	.	.	.	.	.	.	.	.	.	.	.	.	.	.	.	.	.	.	.	.	.	.	.	.	.	.	.
H9	Q	.	.	.	.	.	.	.	.	.	.	.	.	.	.	.	A	.	.	.	.	.	.	C	.	.	.	G	.	.	T	G	.	.	.	.	T	.	.	.	.	.	.	.	.	G	.	.	.	.	.	G	G	.	.	.	.	.	.	.	.	.	.	A	.	.	.	G
H10	B	.	.	.	A	.	.	.	.	.	.	.	.	.	.	.	.	.	.	G	.	.	.	.	.	.	.	.	.	T	.	.	.	T	.	G	.	.	G	.	.	T	.	.	.	.	.	.	.	.	.	.	.	C	.	.	G	.	.	.	.	.	.	.	.	.	.	.
H11	Q	.	.	.	.	.	.	.	.	.	A	.	.	.	.	.	A	.	.	.	.	.	.	C	.	.	A	.	.	.	T	G	.	.	.	.	T	.	.	.	.	.	.	.	.	G	.	.	.	.	.	G	G	.	.	.	.	.	.	.	.	.	.	.	.	.	.	G
H12	L	C	.	G	.	T	.	.	.	.	A	.	.	.	C	C	.	.	G	.	.	.	.	.	.	.	.	.	.	.	T	.	.	.	.	.	.	.	.	.	.	T	T	.	.	G	T	.	.	.	.	.	.	.	.	.	.	.	.	.	.	.	.	.	.	.	.	G
H13	G	.	T	.	.	.	.	.	.	.	.	.	G	.	.	.	.	.	.	G	.	.	.	.	.	.	.	G	T	.	T	.	.	.	.	.	.	.	G	.	.	T	.	.	.	.	.	.	.	.	.	G	.	C	.	.	.	.	.	.	.	.	.	.	.	.	.	.
H14	A	.	.	.	.	.	.	.	.	.	.	.	.	.	C	.	.	.	.	.	.	.	.	.	.	G	.	.	.	.	.	.	.	.	.	.	.	G	.	.	.	T	.	.	.	G	.	.	.	.	.	.	.	.	.	.	.	.	.	.	.	C	C	.	.	.	.	.
H15	D	.	.	.	.	.	.	.	.	.	.	.	.	.	C	.	.	.	.	.	.	.	.	.	.	.	.	.	.	.	.	.	.	.	.	.	.	G	.	.	.	.	.	.	.	.	.	.	.	.	.	.	.	.	.	.	.	.	.	.	.	.	.	.	.	.	.	.
H16	O/P	.	.	.	.	.	.	.	.	.	.	.	.	.	.	.	.	.	.	.	.	.	G	C	.	.	.	.	.	.	T	G	.	.	.	.	.	.	.	.	.	.	.	.	.	G	.	.	.	.	.	.	G	.	.	.	.	.	.	.	.	.	.	A	.	.	.	G
H17	Q	.	.	.	.	.	.	.	.	.	.	.	G	.	.	.	A	.	.	G	.	.	.	C	.	.	A	.	.	.	T	G	.	.	.	.	T	.	.	.	.	.	.	.	.	G	.	.	.	.	.	G	G	.	.	T	.	.	.	.	.	.	.	.	.	.	.	G
H18	A	.	.	.	.	.	.	.	.	.	.	.	.	.	C	.	.	.	.	.	.	.	.	.	.	G	.	.	.	.	.	.	.	.	.	.	.	.	.	.	.	.	.	.	.	.	.	.	.	.	.	.	.	.	.	.	.	.	.	.	.	.	.	.	.	.	.	.
H19	O/P	.	.	.	.	.	.	.	.	.	.	.	.	.	.	.	.	.	.	.	.	.	G	C	.	.	.	.	.	.	T	G	.	.	G	.	.	.	.	.	.	.	.	.	.	G	.	.	.	.	.	.	G	.	.	.	.	.	.	.	.	.	.	A	.	.	.	G
H20	G	.	.	.	.	.	.	T	.	.	.	.	G	.	.	.	.	T	.	G	.	.	.	C	.	.	.	.	.	.	.	.	.	.	.	.	.	.	.	.	.	T	.	.	.	.	.	.	.	.	.	.	.	.	.	.	.	.	.	.	.	.	.	.	.	.	.	.
H21	O/P	.	.	.	.	.	.	.	.	.	.	.	G	.	.	.	.	.	.	.	.	.	.	C	.	.	.	.	T	.	T	G	.	.	G	.	.	.	.	.	.	.	.	.	.	G	.	.	.	.	.	.	G	.	.	.	.	.	.	A	.	.	.	A	.	.	.	G
H22	D	.	.	.	.	.	.	.	.	.	.	.	.	.	C	.	.	.	.	.	.	.	.	.	.	.	.	.	.	.	.	.	.	.	.	.	.	G	.	.	.	.	.	.	.	.	.	.	.	.	.	.	.	.	.	.	.	.	.	A	.	.	.	.	.	.	.	.
H23	O/P	.	.	.	.	.	.	.	.	.	.	.	G	.	.	.	.	.	.	.	.	.	G	C	.	.	.	.	T	.	T	G	.	.	G	.	.	.	.	.	.	.	.	.	.	G	.	.	.	.	.	.	.	.	.	.	.	.	.	A	.	.	.	.	.	.	.	G
H24	O/P	.	.	.	.	.	.	.	.	.	.	.	G	.	.	.	A	.	.	.	.	.	G	C	.	.	.	.	.	.	T	G	.	.	G	.	.	.	.	.	.	.	.	.	.	G	.	.	.	.	.	.	G	.	.	.	.	.	.	.	.	.	.	A	.	.	.	G
H25	L	C	.	G	.	.	.	.	.	.	A	.	.	.	.	C	A	.	.	.	.	.	.	.	.	.	.	.	.	.	T	.	.	.	.	.	.	.	.	.	.	T	T	.	.	G	T	.	.	.	.	.	.	.	.	.	.	.	.	A	.	.	.	.	.	.	.	.
H26	C	.	.	.	.	.	.	.	.	.	.	.	.	.	.	.	.	.	.	G	.	.	.	.	.	.	.	.	.	.	.	.	.	.	.	.	.	G	.	.	.	T	.	.	.	G	T	.	.	.	.	.	.	.	.	.	.	.	.	.	.	.	.	.	.	.	.	.
H27	D	.	.	.	.	.	.	.	.	.	.	.	.	.	C	.	.	.	.	G	.	A	.	.	.	.	.	.	.	.	.	.	.	.	.	.	.	G	.	.	.	.	.	.	.	.	.	.	.	.	.	.	.	.	G	.	.	.	.	.	.	.	.	.	.	.	.	.
H28	D	.	T	.	.	.	.	.	.	.	.	.	.	.	C	.	.	.	.	G	.	.	.	.	.	.	.	.	.	.	.	.	.	.	.	.	.	G	.	.	.	.	.	.	.	.	.	.	.	.	.	.	.	.	G	.	.	.	.	.	.	.	.	.	.	.	.	.
H29	G	.	.	.	.	.	.	T	.	.	.	.	G	.	.	.	.	T	.	G	.	A	.	C	.	.	.	.	.	.	.	.	.	.	.	.	.	.	.	.	.	T	.	.	.	.	.	.	.	.	C	.	.	.	.	.	.	.	.	.	.	.	.	.	.	.	.	.
H30	L	C	.	G	.	T	.	.	.	.	A	.	.	.	.	C	A	.	G	.	.	.	.	.	.	.	.	.	.	.	T	.	.	.	.	.	.	.	.	.	.	T	T	.	.	G	T	.	.	.	.	.	.	.	.	.	.	.	.	.	.	.	.	.	.	.	.	.
H31	A	.	.	.	.	.	.	.	.	.	.	.	.	.	C	.	.	.	.	.	.	.	.	.	.	G	.	.	.	.	.	.	.	.	.	.	.	G	.	.	.	.	.	.	.	.	.	.	.	.	.	.	.	.	.	.	.	.	.	.	.	.	.	.	.	.	.	.
H32	D	.	.	.	.	.	.	.	.	.	.	.	.	.	.	.	.	.	.	G	.	A	.	.	.	.	.	.	.	.	.	.	.	.	.	.	.	G	.	.	.	.	.	.	.	.	.	.	.	.	.	.	.	.	.	.	.	.	.	.	.	.	.	.	.	.	.	.
H33	D	.	.	.	.	.	.	.	.	.	.	.	.	.	C	.	.	.	.	G	.	A	.	.	.	.	.	.	.	.	.	.	.	.	.	.	.	.	.	.	.	.	.	.	.	.	.	.	.	.	.	.	.	.	.	.	.	.	.	.	.	.	.	.	.	.	.	.
H34	D	.	T	.	.	.	.	.	.	.	.	.	.	.	C	.	.	.	.	.	.	A	.	.	.	.	.	.	.	.	.	.	.	.	.	.	.	G	.	.	.	.	.	.	.	.	.	.	.	.	.	.	.	.	G	.	.	.	.	.	.	.	.	.	.	.	.	.
H35	N	.	.	.	.	.	.	.	.	.	A	.	.	C	.	.	.	.	.	.	.	.	.	.	.	.	.	.	.	.	T	.	T	.	.	.	.	.	G	T	T	.	.	.	.	G	.	.	.	C	.	.	.	.	.	.	.	.	.	.	.	.	.	A	C	.	.	G
H36	C	.	T	.	.	.	.	.	.	.	.	.	.	.	C	.	.	.	.	G	.	.	.	.	.	.	.	.	.	.	.	.	.	.	.	.	.	G	.	.	.	T	.	.	.	G	T	.	.	.	.	.	.	.	.	.	.	.	.	.	.	.	.	.	.	.	.	.
H37	D	C	.	.	.	.	.	.	.	.	.	.	.	.	C	.	.	.	.	G	.	A	.	.	.	G	.	.	.	.	.	.	.	.	.	.	.	.	.	.	.	.	.	.	.	.	.	.	.	.	.	.	.	.	G	.	.	.	.	.	.	.	.	.	.	.	.	.
H39	D	.	.	.	.	.	.	.	.	.	.	.	.	.	C	.	.	.	.	G	.	A	.	.	.	.	.	.	.	.	.	.	.	.	.	.	.	.	.	.	.	.	.	.	.	.	.	.	.	.	.	.	.	.	G	.	.	.	.	.	.	.	.	.	.	.	.	.
H40	G	.	.	.	.	.	.	T	.	.	.	.	G	.	.	.	.	T	.	G	.	A	.	.	.	.	.	.	.	.	.	.	.	.	.	.	.	.	.	.	.	T	.	.	.	.	.	.	.	.	.	.	.	.	.	.	.	.	.	.	.	.	.	.	.	.	.	.
H41	O/P	.	.	.	.	.	.	.	.	.	.	.	G	.	.	.	A	.	.	.	.	.	G	.	.	G	.	.	.	.	T	G	.	.	G	.	.	.	.	.	.	.	.	.	.	G	.	.	.	.	.	.	G	.	.	.	.	.	.	.	.	.	.	A	.	.	.	G
H42	O/P	.	.	.	.	.	.	.	.	.	.	.	G	.	.	.	A	.	.	.	.	.	G	C	.	.	.	.	.	.	T	G	.	.	G	.	.	.	.	.	.	.	.	.	.	G	.	.	.	.	.	.	G	.	.	.	.	.	.	.	.	.	.	.	.	.	.	G
H43	G	.	.	.	.	.	.	T	.	.	.	.	G	.	.	.	.	T	.	G	.	A	.	C	.	.	.	.	.	.	.	.	.	.	.	.	.	.	.	.	.	T	.	.	.	.	.	.	.	.	C	.	.	.	.	.	.	.	.	A	.	.	.	.	.	.	.	.
H44	C	.	.	.	.	.	.	.	.	.	.	.	.	.	.	.	.	.	.	G	.	.	.	.	.	.	.	.	.	.	.	.	.	.	.	.	.	G	.	.	.	T	.	.	.	G	.	.	.	.	.	.	.	.	.	.	.	.	.	.	.	.	.	.	.	.	.	.
H45	I	.	.	.	.	.	G	.	.	.	A	.	.	.	.	.	.	.	.	G	.	.	.	.	T	.	.	.	.	.	T	.	.	.	.	.	.	G	.	.	.	T	.	.	.	.	T	.	.	.	.	.	.	.	.	.	.	.	.	.	.	.	.	.	.	.	.	.
H46	O/P	.	.	.	.	.	.	.	.	.	.	.	G	.	.	.	A	.	.	.	.	.	G	C	.	.	.	.	.	.	T	G	.	.	G	.	.	.	.	.	.	.	.	.	.	G	.	G	.	.	.	.	G	.	.	.	.	.	.	.	.	.	.	A	.	.	.	G
H47	G	.	.	.	.	.	.	T	.	.	.	.	G	.	.	.	.	T	.	G	.	.	.	.	.	.	.	.	.	.	.	.	.	.	.	.	.	.	.	.	.	T	.	.	.	.	.	.	.	.	.	.	.	.	.	.	.	.	.	.	.	.	.	.	.	.	.	.
H48	O/P	.	.	.	.	.	.	.	.	.	.	.	G	.	.	.	A	.	.	.	.	.	G	C	.	.	.	.	.	.	T	G	.	.	G	.	.	.	.	.	.	.	.	.	.	G	.	.	.	.	.	.	G	.	.	.	.	.	.	.	.	.	.	.	.	.	C	G
H49	Q	.	.	.	.	.	.	.	A	T	A	.	.	.	.	.	A	.	.	.	.	.	.	C	.	.	A	G	.	.	T	.	.	.	.	.	T	.	.	.	.	.	.	.	.	G	.	.	.	.	.	G	G	.	.	.	.	.	.	.	.	.	.	A	.	.	.	G
H50	I	.	.	.	.	.	G	.	.	.	.	G	.	.	.	.	.	.	.	G	.	.	.	.	T	.	.	.	.	.	T	.	.	.	.	.	.	G	.	.	.	T	.	.	.	.	T	.	.	.	.	.	.	.	.	.	.	.	T	.	.	.	.	.	.	.	.	.
H51	O/P	.	.	.	.	.	.	.	.	.	.	.	G	.	.	.	A	.	.	.	.	.	G	C	.	.	.	.	.	.	T	G	.	.	G	.	.	.	.	.	.	.	.	.	.	G	.	.	.	.	.	.	G	.	.	.	.	.	.	.	.	.	.	A	.	.	.	.
H52	O/P	.	.	.	.	.	.	.	.	.	.	.	G	.	.	.	A	.	.	.	.	.	G	C	.	.	.	.	.	.	T	G	.	.	G	.	.	.	.	.	.	.	.	.	.	G	.	.	.	.	.	.	G	.	G	.	.	.	.	.	.	.	.	A	.	.	C	G
H53	L	C	.	G	.	T	.	.	.	.	A	.	.	.	.	C	A	.	G	.	.	.	.	.	.	.	.	.	.	.	.	.	.	.	.	.	.	.	.	.	.	T	T	.	.	G	T	.	.	.	.	.	.	.	.	.	.	.	.	.	.	.	.	.	.	C	.	G
H54	O/P	.	.	.	.	.	.	.	.	.	.	.	G	.	.	.	A	.	.	.	.	.	G	C	.	G	.	.	.	.	T	G	.	.	G	.	.	.	.	.	.	.	.	.	.	G	.	.	.	.	.	.	G	.	.	.	.	.	.	.	.	.	.	A	.	.	.	G
H55	L	C	.	G	.	T	.	.	.	.	A	.	.	.	.	C	A	.	G	.	.	.	.	.	.	G	.	.	.	.	T	.	.	.	.	.	.	.	.	.	.	T	T	.	.	G	T	.	.	.	.	.	.	.	.	.	.	.	.	.	.	.	.	.	.	C	.	G
H56	L	C	.	G	.	T	.	.	.	.	A	.	.	.	.	C	A	.	G	.	.	.	.	.	.	.	.	.	.	.	T	.	.	.	.	.	.	.	.	.	.	T	T	.	.	G	T	.	.	.	.	.	.	.	.	.	.	.	.	.	.	.	.	.	.	C	.	G
H57	O/P	.	.	.	.	.	.	.	.	.	A	.	G	.	.	.	A	.	.	.	.	.	G	C	.	.	.	.	.	.	T	G	.	.	.	.	.	.	.	.	.	.	.	.	.	G	.	.	.	.	.	.	.	.	.	.	.	.	.	.	.	.	.	A	.	.	.	G
H58	I	.	.	.	.	.	G	.	.	.	A	.	.	.	.	.	.	.	.	.	.	.	.	.	T	.	.	.	.	.	T	.	.	.	.	.	.	G	.	.	.	.	.	.	.	.	T	.	.	.	.	.	.	.	.	.	.	.	.	.	.	.	.	.	.	.	.	.
H59	D	.	.	.	.	.	.	.	.	.	.	.	.	.	C	.	.	.	.	G	.	A	.	.	.	.	.	.	.	.	.	.	.	.	.	.	.	G	.	.	.	.	.	.	T	.	.	.	.	.	.	.	.	.	G	.	.	.	.	.	.	.	.	.	.	.	.	.
H60	L	C	.	G	.	T	.	.	.	.	A	.	.	.	C	C	.	.	.	.	.	.	.	.	.	.	.	.	.	.	.	.	.	.	.	.	.	.	.	.	.	T	T	.	.	G	T	.	.	.	.	.	.	.	.	.	.	.	.	.	.	.	.	.	.	.	.	G
H61	I	.	.	.	.	.	G	.	.	.	.	.	.	.	.	.	.	.	.	.	.	.	.	.	T	.	.	.	.	.	T	.	.	.	.	.	.	G	.	.	.	.	.	.	.	.	T	.	.	.	.	.	.	.	.	.	.	.	.	.	.	.	.	.	.	.	.	.
H62	I	.	.	.	.	.	G	.	.	.	A	.	.	.	.	.	.	.	.	.	.	.	.	.	.	.	.	.	.	.	T	.	.	.	.	.	.	G	.	.	.	.	.	.	.	.	T	.	.	.	.	.	.	.	.	.	.	.	.	.	.	.	.	.	.	.	.	.
H63	Q	.	.	.	.	.	.	.	.	.	.	.	.	.	.	.	A	.	.	.	C	.	.	C	.	.	A	G	.	.	T	G	.	.	.	.	T	.	.	.	.	.	.	.	.	G	.	.	.	.	.	G	G	.	.	.	.	.	.	.	.	.	.	A	.	.	.	G
H64	I	C	.	.	.	.	G	.	.	.	A	.	.	.	.	.	.	.	.	.	.	.	.	.	T	.	.	.	.	.	T	.	.	T	.	.	.	G	.	.	.	.	.	.	.	.	T	.	.	.	.	.	.	.	.	.	.	.	.	.	.	.	.	.	.	.	.	.
H65	I	.	.	.	.	.	G	.	.	.	A	.	.	.	.	.	.	.	.	G	.	.	.	.	T	.	.	.	.	.	T	.	.	.	.	.	.	G	.	.	.	T	.	.	.	.	T	.	.	.	.	.	.	.	.	.	.	.	T	.	.	.	.	.	.	.	.	.
H66	L	C	.	G	.	T	.	.	.	.	.	.	.	.	.	C	.	.	G	.	.	.	.	.	.	.	.	.	.	.	T	.	.	.	.	.	.	.	.	.	.	T	T	T	.	G	T	.	.	.	.	.	.	.	.	.	.	.	.	.	.	.	.	.	.	.	.	G
H67	L	C	.	G	.	T	.	.	.	.	A	.	.	.	.	C	.	.	G	.	.	.	.	.	.	.	.	.	.	.	.	.	.	.	.	.	.	.	.	.	.	.	.	.	.	G	T	.	.	.	.	.	.	.	.	.	.	.	.	.	.	.	.	.	.	C	.	G
H68	Q	.	.	.	.	.	.	.	.	.	A	.	.	.	.	.	A	.	.	.	.	.	.	C	.	.	.	G	.	.	T	G	.	.	.	.	T	.	.	.	.	.	.	.	.	G	.	.	G	.	.	G	G	.	.	.	.	.	.	.	.	.	.	A	.	.	.	G
H69	Q	.	.	.	.	.	.	.	.	.	.	.	G	.	.	.	A	.	.	.	.	.	.	C	.	.	.	G	.	.	T	G	.	.	.	.	T	.	.	.	.	.	.	.	.	.	.	.	G	.	.	G	G	.	.	.	.	.	.	.	.	.	.	A	.	.	.	G
H70	L	C	.	G	.	T	.	.	.	.	.	.	.	.	.	C	.	.	G	.	.	.	.	.	.	.	.	.	.	.	T	.	.	.	.	.	.	.	.	.	.	T	T	.	.	G	T	.	.	.	.	.	.	.	.	.	G	.	.	.	.	.	.	.	.	.	.	G
H71	L	.	T	G	.	T	.	.	.	.	A	.	.	.	.	C	.	.	G	.	.	.	.	.	.	.	.	.	.	.	T	.	.	.	.	.	.	.	.	.	.	T	T	.	.	G	T	.	.	.	.	.	.	.	.	.	.	.	.	.	.	.	.	.	.	C	.	G
H72	C	.	.	.	.	.	.	.	.	.	.	.	.	.	C	.	.	.	.	G	.	.	.	.	.	.	.	.	.	.	.	.	.	.	.	.	.	G	.	.	.	T	.	.	.	G	T	.	.	.	.	.	.	.	.	.	.	.	.	.	.	.	.	.	.	.	.	.
H73	Q	.	.	.	.	.	.	.	.	.	.	.	G	.	.	.	A	.	.	.	.	.	.	C	.	.	.	G	.	.	T	G	.	.	.	.	T	.	.	.	.	.	.	.	.	G	.	.	G	.	.	G	G	.	.	.	.	.	.	.	.	.	.	A	.	.	.	G
H74	Q	.	.	.	.	.	.	.	.	.	A	.	.	.	.	.	A	.	.	.	.	.	.	C	.	.	.	.	.	.	T	G	.	.	.	.	T	.	.	.	.	.	.	.	.	G	.	.	G	.	.	G	G	.	.	.	.	.	.	.	.	.	.	A	.	.	.	G
H75	G	.	.	.	.	.	.	T	.	.	.	.	G	.	.	.	.	T	.	G	.	A	.	C	.	.	.	.	.	.	.	.	.	.	.	.	.	.	.	.	.	T	.	.	.	.	.	.	.	.	.	.	.	.	.	.	.	.	.	.	.	.	.	.	.	.	.	.
H76	Q	.	.	.	.	.	.	.	.	.	A	.	.	.	.	.	A	.	.	.	.	.	.	C	.	.	.	G	.	.	T	G	.	.	.	.	T	.	.	.	.	.	.	.	.	G	.	.	G	.	.	G	G	.	.	.	.	.	.	.	.	.	.	.	.	.	.	G
H77	L	C	.	G	.	.	.	.	.	.	.	.	.	.	.	C	.	.	G	.	.	.	.	.	.	.	.	.	.	.	T	.	.	.	.	.	.	.	.	.	.	T	T	.	.	G	T	.	.	.	.	.	.	.	.	.	G	.	.	.	.	.	.	.	.	C	.	G
H78	I	.	.	.	.	.	.	.	.	.	A	.	.	.	.	.	.	.	.	G	.	.	.	.	T	.	.	.	.	.	T	.	.	.	.	.	.	G	.	.	.	.	.	.	.	.	T	.	.	.	.	.	.	.	.	.	.	.	T	.	.	.	.	.	.	.	.	.
H79	L	C	.	G	.	T	.	.	.	.	.	.	.	.	.	C	.	.	G	.	.	.	.	.	.	.	.	.	.	.	T	.	.	.	.	.	.	.	.	.	.	T	T	.	.	G	.	.	.	.	.	.	.	.	.	.	.	.	.	.	.	.	.	.	.	.	.	G
H80	Q	.	.	.	.	.	.	.	.	.	.	.	G	.	.	.	A	.	.	.	.	.	.	C	.	.	A	G	.	.	T	.	.	.	.	.	T	.	.	.	.	.	.	.	.	G	.	.	.	.	.	G	G	.	.	.	.	.	.	.	.	.	.	A	.	.	.	G
H81	D	.	.	.	.	.	.	.	.	.	A	.	.	.	C	.	.	.	.	G	.	A	.	.	.	.	.	.	.	.	.	.	.	.	.	G	.	G	.	.	.	.	.	.	.	.	.	.	.	.	.	.	.	.	G	.	.	.	.	.	.	.	.	.	.	.	.	.
H82	O/P	.	.	.	.	.	.	.	.	.	.	.	G	.	.	.	A	.	.	.	.	.	G	C	.	.	.	.	.	.	T	G	.	.	G	.	.	.	.	.	.	.	.	.	.	G	.	.	.	.	.	.	G	.	.	.	.	.	.	.	G	.	.	A	.	.	.	.
H83	L	C	.	G	.	T	.	.	.	.	A	.	.	.	.	C	.	.	G	.	.	.	.	.	.	.	.	.	.	.	T	.	.	.	.	.	.	.	.	.	.	T	T	.	.	G	T	.	.	.	.	.	.	.	.	.	.	.	.	.	.	.	.	.	.	.	.	G
H84	M	.	.	.	.	.	.	.	.	.	.	.	.	.	.	.	.	.	.	.	C	.	.	.	.	.	.	.	.	.	T	.	T	.	.	.	.	.	G	.	T	.	.	.	.	G	.	.	.	C	.	.	.	.	.	.	.	.	.	.	.	.	.	A	C	.	.	G
H85	L	.	.	.	.	.	.	.	.	.	A	.	.	.	.	C	A	.	G	.	.	.	.	.	.	.	.	.	.	.	T	.	.	.	.	.	.	.	.	.	.	T	T	.	.	G	.	.	.	.	.	.	.	.	.	.	.	.	.	A	.	.	.	.	.	.	.	.
H86	Q	.	.	.	.	.	.	.	.	.	.	.	.	.	.	.	A	.	.	.	.	.	.	.	.	.	.	G	.	.	.	.	.	.	.	.	T	.	.	.	.	.	.	.	.	G	.	.	G	.	.	G	G	.	.	.	.	.	.	.	.	.	.	A	.	.	.	G
H87	L	C	.	G	.	T	.	.	.	.	A	.	.	.	.	C	.	.	G	.	.	.	.	.	.	.	.	.	.	.	T	.	.	.	.	.	.	.	.	.	.	T	T	.	.	G	T	.	.	.	.	.	.	.	.	.	.	.	.	.	.	.	.	.	.	C	.	G
H88	I	.	.	.	.	.	G	.	.	.	.	.	.	.	.	.	.	.	.	G	.	.	.	.	T	.	.	.	.	.	T	.	.	.	.	.	.	G	.	.	.	T	.	.	.	.	T	.	.	.	.	.	.	.	.	.	.	.	T	.	.	.	.	.	.	.	.	.
H89	I	.	.	.	.	.	G	.	.	.	A	.	G	.	.	.	.	.	.	G	.	.	.	.	T	.	.	.	.	.	T	.	.	.	.	.	.	G	.	.	.	.	.	.	.	.	.	.	.	.	.	.	.	.	.	.	.	.	.	.	.	.	.	.	.	.	.	.
H90	D	C	.	.	.	.	.	.	.	.	A	.	.	.	C	.	.	.	.	G	.	A	.	.	.	.	.	.	.	.	.	.	.	.	.	G	.	G	.	.	.	.	.	.	.	.	.	.	.	.	.	.	.	.	G	.	.	.	.	.	.	.	.	.	.	.	.	.
H91	O/P	.	.	.	.	.	.	.	.	.	.	.	G	.	.	.	A	.	.	.	.	.	G	C	.	.	.	.	.	.	T	G	.	.	G	.	.	.	.	.	.	.	.	.	.	G	.	.	.	.	.	.	.	.	.	.	.	.	.	.	.	.	.	A	.	.	.	G
H93	Q	.	.	.	.	.	.	.	.	.	A	.	.	.	.	.	A	.	.	.	.	.	.	C	.	.	.	G	.	.	T	G	.	.	.	.	T	.	.	.	.	.	.	.	.	G	.	.	G	.	.	.	.	.	.	.	.	.	.	.	.	.	.	A	.	.	.	G
H94	C	.	.	.	.	.	.	.	.	.	.	.	.	.	.	.	.	.	.	.	.	.	.	.	.	.	.	.	.	.	.	.	.	.	.	.	.	G	.	.	.	T	.	.	.	.	T	.	.	.	.	.	.	.	.	.	.	.	.	.	.	.	.	.	.	.	.	.
H95	C	.	.	.	.	.	.	.	.	.	.	.	G	.	.	.	.	.	.	G	.	.	.	.	.	.	.	.	.	.	.	.	.	.	.	.	.	G	.	.	.	T	.	T	.	G	T	.	.	.	.	.	.	.	.	.	.	.	.	.	.	.	.	.	.	.	.	.
H96	G	.	.	.	.	.	.	T	.	.	A	.	G	.	.	.	.	T	.	G	.	A	.	.	.	.	.	.	.	.	.	.	.	.	.	.	.	.	.	.	.	T	T	.	.	.	.	.	.	.	.	.	.	.	.	.	.	.	.	.	.	.	.	.	.	.	.	.

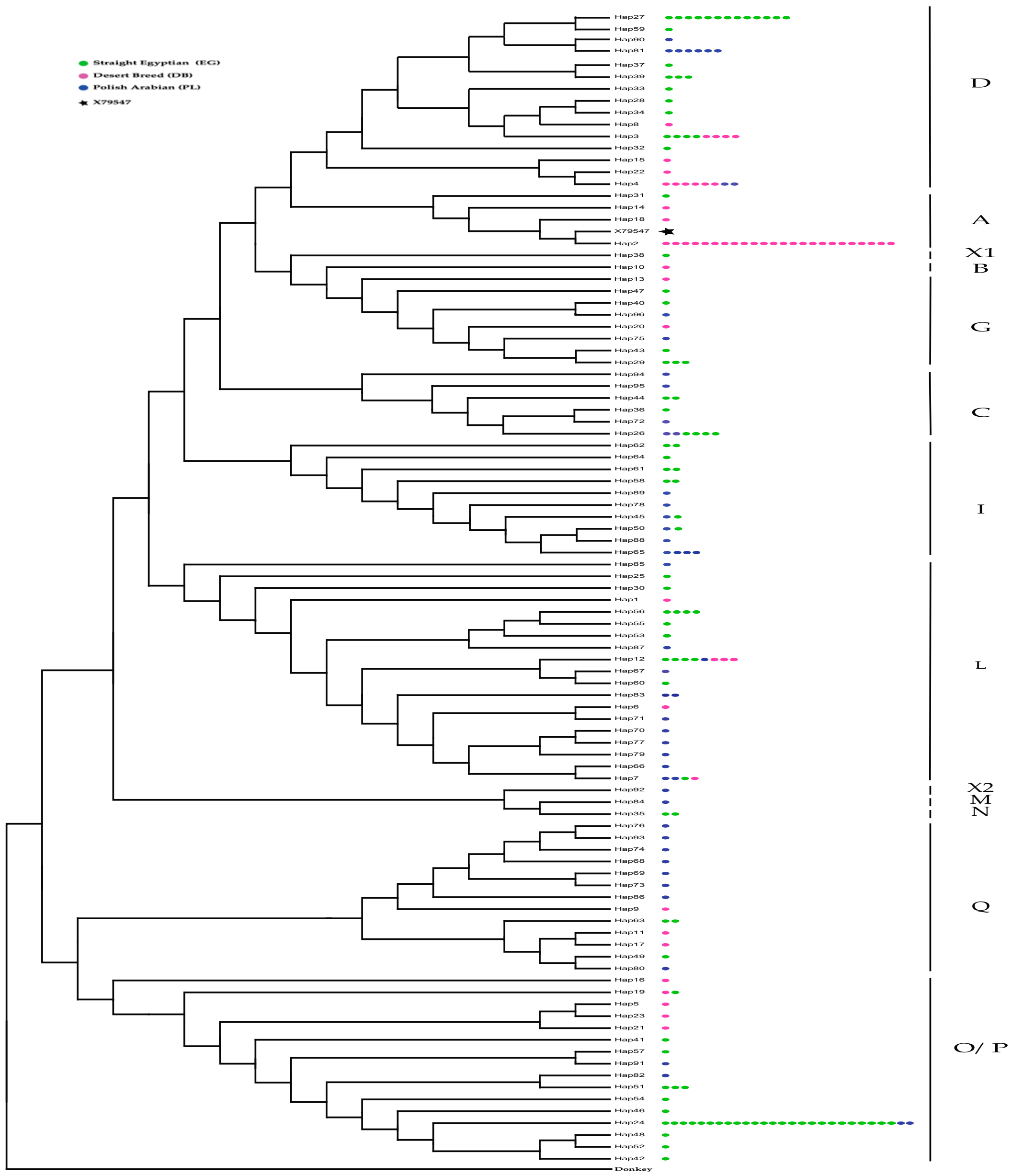

Supplementary Figure S2 : Consensus Neighbo r-joining tree of 96 mtDNA haplotypes found in arabian horse populations . The t ree was drawn based upon 1000 bootstrap replicates and the values we re all below 50. The reference donkey sequence nc 0017788 was used as an out-g roup. The individuals with each haplotype a re represented by colo red circles depending on population. Populations details a re represented in Table 1.

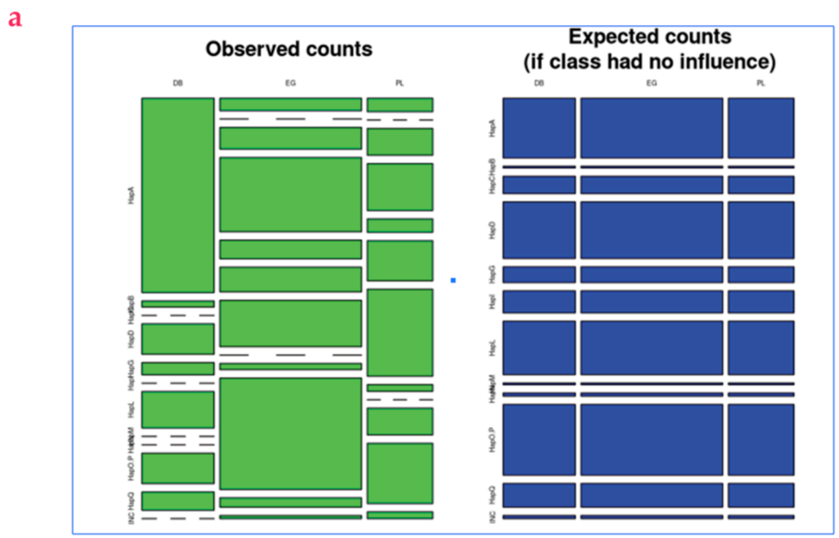

Interpretations
For the population : P-value is less than 0.05, so the Desert bred (DB), Straight Egyptian (EG) and Polish Arabian populations are different in term of haplogrous frequencies.

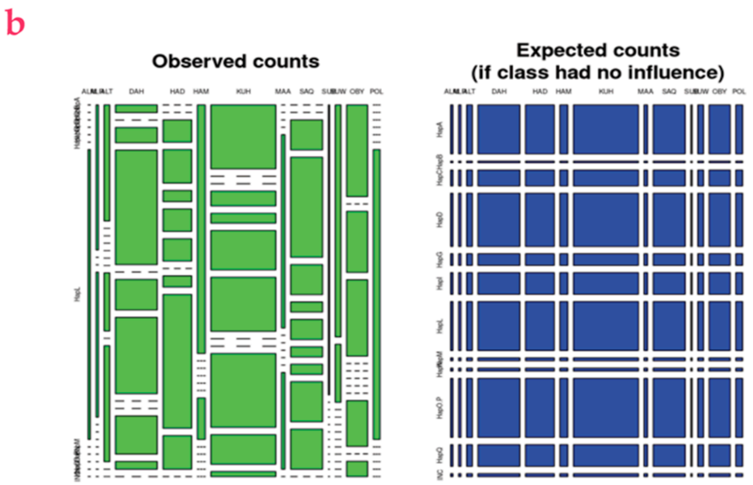

Interpretations
For the strians : P-value is less than 0.05, so the strains are different in term of haplogrous frequencies.

Supplementary Figure S3 : Chi-2 test results of haplogroups among both populations (a) and strains (b)

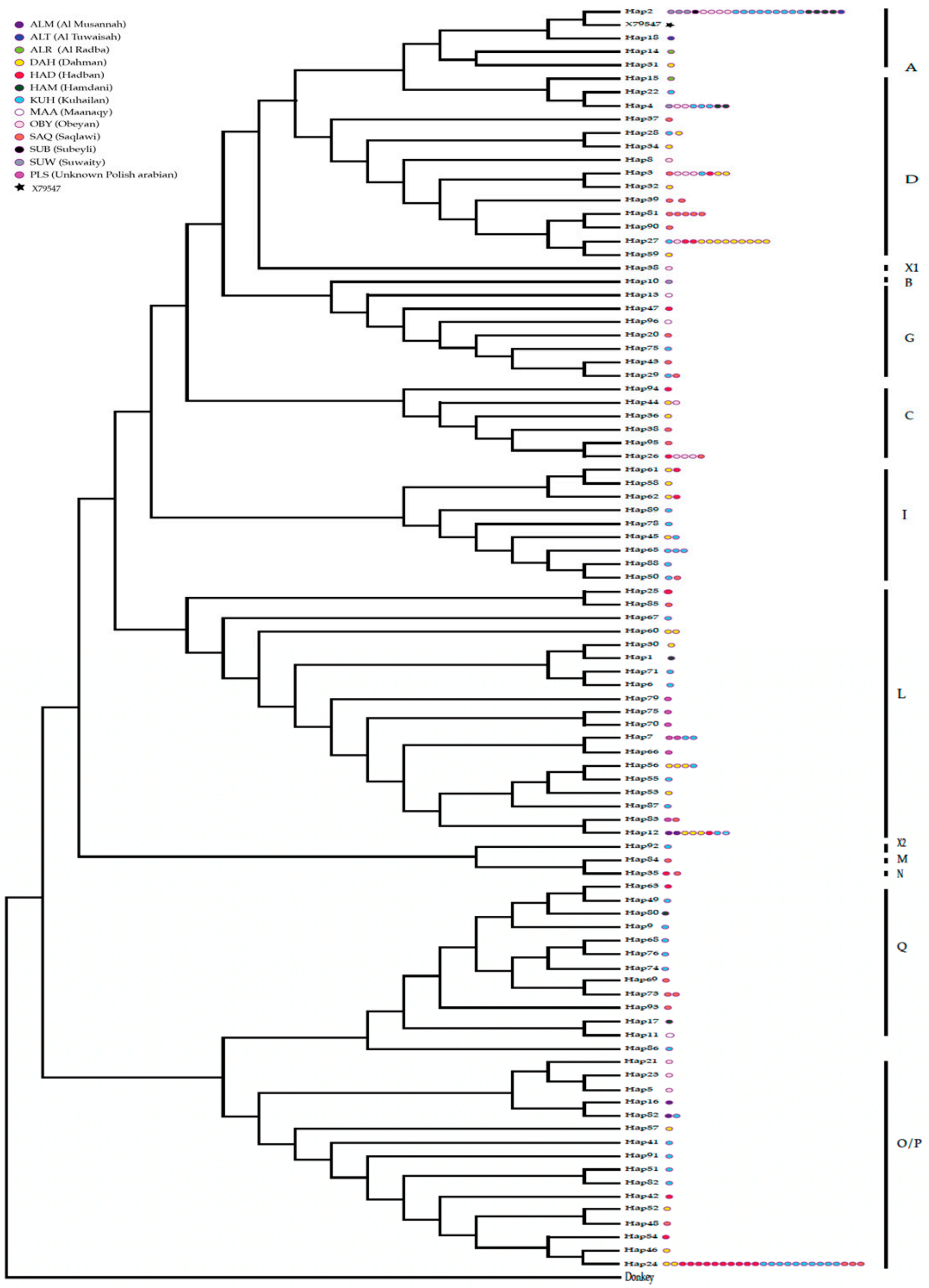

Supplementary Figure S4 . Consensus Neighbor-joining tree of the 93 mitochondrial haplotypes found in Arabian horses strains. The reference sequence GenBank X79547. The reference donkey sequence nc 0017788 was used as an out-group. The individuals with each haplotype are represented by colored circles depending on strains. The tree was drawn based upon 1000 bootstrap replicates and the values were all below 50.

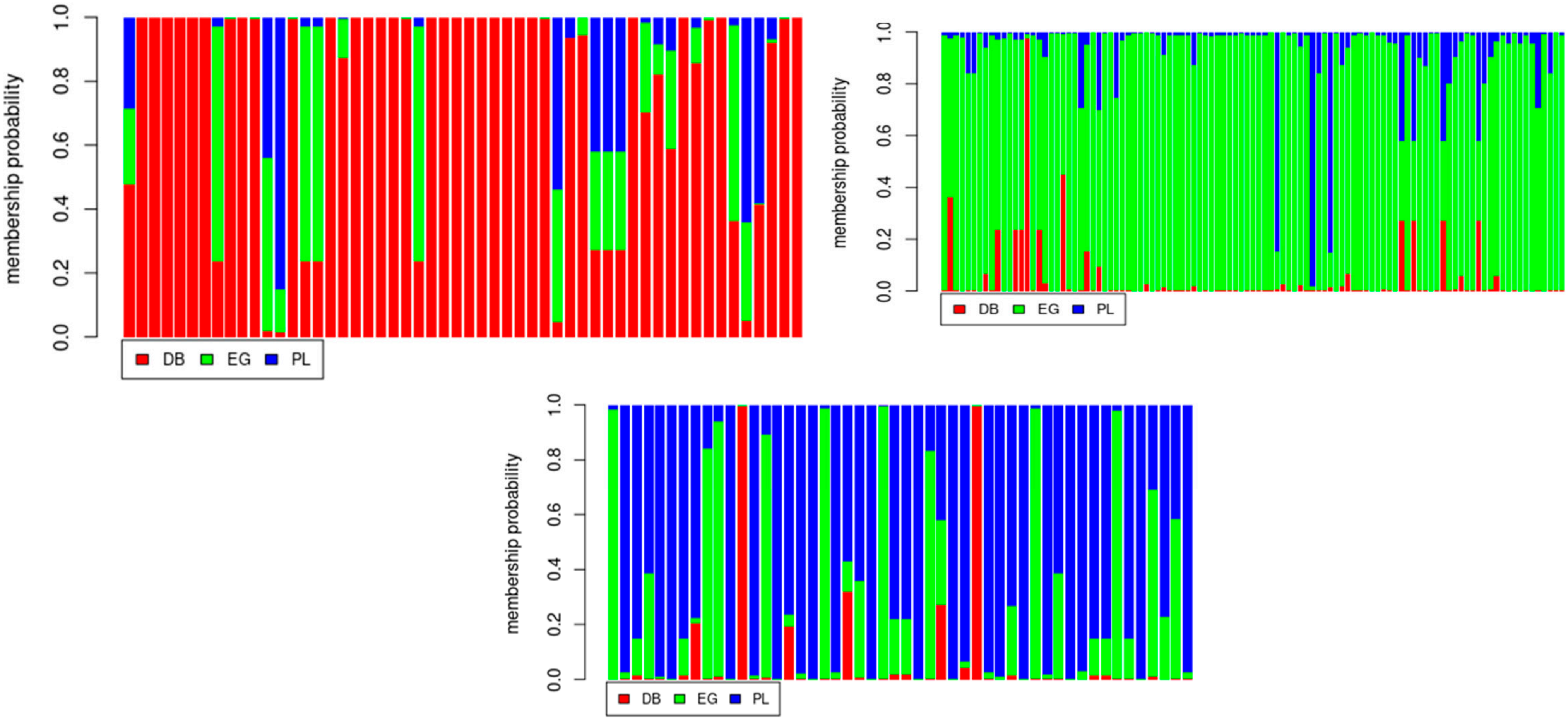

Figure S5: Probability of membership for each individual in the studied populations. Each column is representative of one individual and each population is shown by a separate color.

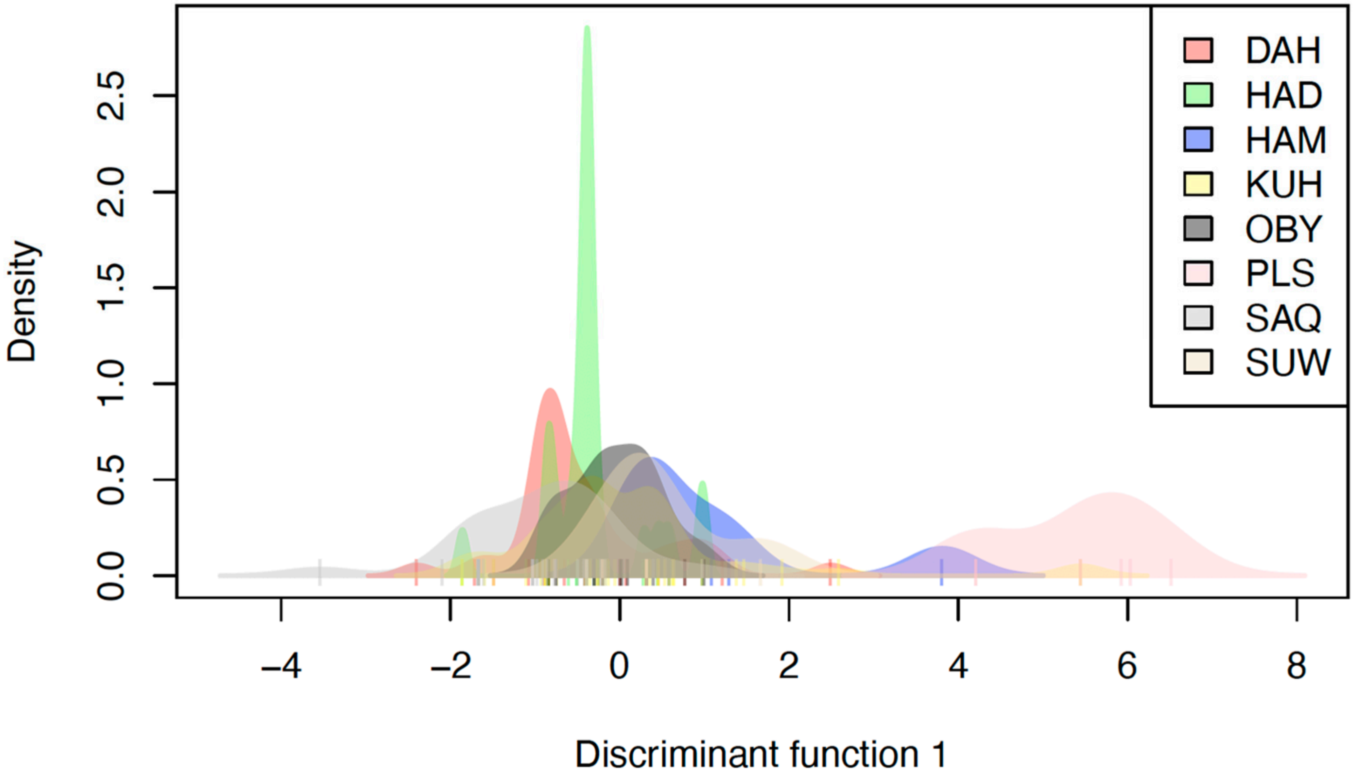

Figure S6: Density plot of Arabian horse strain individuals based on first discriminant function.
